# Observations on Aneurism, Selected from the Works of the Principal Writers on That Disease, from the Earliest Periods to the Close of the Last Century

**Published:** 1845-04

**Authors:** 


### Observations on Aneurism, selected from the Works of the principal Writers on that Disease from the earliest Periods to the Close of the last Century. Translated and Edited by *John E. Erichsen*, Lecturer on General Anatomy and Physiology at the Westminster Hospital. London, 1844. 8vo. pp. 524. Printed for the Sydenham Society.

We cannot much congratulate the Members of the Sydenham Society on the selection which their Council has made upon the present occasion. That a volume, consisting chiefly of memoirs, and extracts from memoirs, of the old surgical writers on a subject, that was so imperfectly and inaccurately understood by them as aneurism, should have been chosen—while so very few classic works of medical literature have yet appeared from their press—has surprised and disappointed us not a little. How is it that we hear nothing of a new edition of the writings of Harvey for example, or of Morgagni, or of Boerhaave—not to make any mention of those of Hippocrates and the fathers of our science—being even under the consideration of the managing Committee ?

It can serve little good to collect together the opinions of writers on a disease which it was utterly impossible for them to have any correct notions upon, while the cardinal fact of the circulation of the blood was yet unknown. Such at least is the opinion that we have come to, after a careful perusal of the present volume. No one will be so hardy, we should think, as to dispute the immeasureable superiority of modern over ancient surgery. The one is a science, the other was (on too many occasions) a rude and barbarous art. It could not be otherwise; while so many of the very elementary truths of Physiology were yet unknown, and Pathological Anatomy had not so much as received a name. Not so with Medicine—using this term in contradistinction to Surgery. The course and progress of very many diseases may be accurately portrayed; their origin and causes may be traced out; the various circumstances that affect and modify their symptoms may be detected; and the influence of external agencies and of remedial means may be carefully watched and faithfully described, although the physician is most imperfectly acquainted with the structure of the body. But it is very different with surgical maladies, and more especially with those, that may require a cutting operation for their cure. By these remarks we do not mean to find the very slightest fault with the manner in which Mr. Erichsen has discharged the task committed to his care. He has done all for the subject that it admitted of. We only regret that his industry and scholarship have not been applied to the investigation of another theme. His volume will be useful to the surgical antiquarian, as the " Bibliography," appended to it, " contains a reference to all memoirs and detached essays of any value that have been published on the subject of Aneurism within the period to which the work is confined"—from the time of Galen, who wrote in the second century, down to the closing year of the last one.

In the following article, we purpose to direct our attention (and we hope that of our readers also) more particularly to a notice of the operations introduced and first practised by Anel and by John Hunter, with the view of shewing how deeply the science of surgery is indebted, on this score alone (not to mention others), to our great countryman. It may add to the interest of the enquiry to take a very brief review of the opinions that were held, as to the nature and treatment of the disease under consideration, by the leading surgeons anterior to the time of Anel, and by those also who lived between his time and that of Hunter.

Galen informs us that " an aneurism is a dilatation or relaxation of a venous vessel, or a dispersion of the spirituous matter under the flesh, where it diffuses and distributes itself by jerks (per dissultationem.)"

Aëtius, three or four centuries later, describes the disease in these words:

" A dilatation of the vessels, which the Greeks call an aneurism, may occur in any part of the body, but is most frequently met with in the throat, where it gives rise to a tumour that goes by the name of bronchocele. It very commonly happens to women during parturition, on account of the forcible detention of the spirits." 4.

Skipping over only a thousand years, or thereabouts, we come to the time of Fernelius, who wrote a work *de Morbis Universalibus et Particularibus.* According to him, (A.D. 1542) " an Aneurism is the dilatation of an artery full of spirituous blood." Paré, the pride of French surgery in his day (1582), was of opinion that the disease " is occasioned by the blood and spirits being effused under the flesh." Sennertus (1628) seems to have had more correct views as to the nature of the disease. " It is not to be supposed," says he, " that an Aneurism is occasioned by a dilatation of both coats of an artery, but probably of one only: for arteries possess a double coat; an external one, which is thin, fine, and soft, having many straight fibres, but few oblique, and no transverse ones; and an internal one, which is thick, dense, and hard, having transverse, but no straight or oblique, fibres. And therefore if the internal hard membrane be either ruptured, in consequence of over-stretching, or be opened by a wound, it will not, on account of its hardness, coalesce readily; but as the external one is softer, it readily coalesces; and because it is softer, and has neither oblique nor transverse fibres, it will be distended by the blood and vital spirits which are urgently seeking to escape; and thus this kind of tumour is formed, in which the impetus of the blood and of the vital spirit can actually be seen."

This writer subsequently remarks, with singular sagacity, that " the proximate cause of an aneurism is an opening in the internal tunic, and a dilatation of the external one." Wiseman (1676) combated this opinion of Sennertus, contending that the disease was invariably produced by " the blood bursting quite through the artery into the interstices of the muscles." Among the inducing causes, he enumerates an acrimonious state of the blood itself, " which, being too sharp or thin, erodes the vessel; or, being highly fermented by other causes, bursts through all."

Upwards of 60 pages are devoted by Mr. Erichsen to the observations of Lancisi—*de aneurysmatibus opus posthumum, Romæ,* 1728. We have been unable to detect a single useful remark among them all. Not a few of his cases of aneurism are merely examples of nervous palpitation of some of the large blood-vessels; and, indeed, one of the sections is headed, "on aneurisms that are occasioned by an ichorous eroding principle in hypochondriacal, scorbutic, and hysterical persons." He subsequently remarks, that "all chemists agree in this, that aneurisms may very readily be occasioned by mercurial inunctions. Thus Paré, Ballonius, and others have indubitably proved it to us by the examples they have adduced."

Many cases of aneurism were supposed to be owing to the syphilitic disease: but the most frequent cause (according to Lancisi) is excess in eating and drinking—the blood becoming thereby not only redundant in quantity, but also of an acrid and irritating nature. One of his observations really does deserve notice from its practical importance. "A knowledge of these is especially necessary, not only because aneurism of the cavities and of the vessels of the heart is a more common disease than the majority of medical men have hitherto thought on account of the small number of dissections performed by them, but more particularly because the hidden causes of many diseases are to be found in a dilatation or obstruction of the cavities of the heart. For, indeed, frequent dissections have proved to me that many suffocating asthmatic affections, obstinate oppressions, and frequent palpitations about the heart, dropsy of the chest, and more especially sudden deaths, depend upon one cause, namely, unequal and sudden dilatation of the vessels of the heart."

Let us now briefly notice the Treatment of the Disease that was adopted by the writers whom we have quoted. Aëtius (in the sixth century) recommended, in brachial aneurisms, that the brachial artery be exposed about the middle of the arm, tied with two ligatures, and divided midway between them. The aneurismal sac was then to be laid open and emptied of its contents; after which, the artery, whence the blood flowed, was to be sought for, tied, and cut across.

Paulus Ægineta seems not to have laid open the sac at all. The description of his operation runs thus. After the vessel has been exposed, "a needle being then passed under it, the artery is to be tied with a double ligature, having previously been punctured in the middle; suppuration must then be promoted until the ligatures fall out. But if the aneurism is occasioned by the rupture of the artery, all that can be done is to take it up, together with the skin, between the fingers, and then to pass a needle, armed with a double thread, below the part that is to be included in the ligature. After the needle has been passed through, the noose of the string is to be cut off with a pair of scissors, and thus the tumour, being included between two ligatures, is to be tied on both sides like a staphyloma."

Paré gives the following judicious advice:—

"I would advise the young surgeon not to open aneurisms, provided they be not very small, and situated in parts that are not very important; in which case he may divide the skin above them, and, separating the artery, pass a seton-needle armed with a strong thread under it, and allow the ligature to fall of itself. Nature will then generate flesh, which will block up the artery." 185.

It does not however appear that either he or any of his cotemporaries followed this plan in their practice. Some used compression; others the actual cautery; and, if a cutting operation was resorted to, this consisted in laying open the aneurismal sac (a tourniquet having previously been applied), clearing out its contents, and then endeavouring to tie its upper end. As we might expect, the patient generally died either from hæmorrhage, or from gangrene of the limb supervening.

The following extract from the writings of Morel (1681) is curious from the allusion to the *then* high state of surgery.

"Since the successful employment of ligatures of the vessels in amputation of the members has been generally recognized, most authors have advised the same practice in the operation for aneurism; but with this reserve, that they have considered it useless, or even dangerous, for the large arteries; which has so intimidated many surgeons, that even at present, when surgery appears to have attained the very summit of perfection, many of the most celebrated have abandoned the ligature for a button of vitriol, for the graduated compress, for the constant application of the finger, or for some similar means." 204.

We pass on to the year 1710, when Anel performed his operation of tying the brachial artery, immediately above the aneurismal swelling, without opening its cavity, or in any way interfering with it. The patient, it would seem, was in imminent danger of death from hæmorrhage. As this case may certainly be regarded as unquestionably the most interesting one in the history of the treatment of aneurisms, prior to the time of John Hunter, it may be interesting to mention briefly some of the particulars connected with it. The operation was performed at Rome, on the 30th of January, 1710. The following is Anel's own description of the mode in which it was performed.

"Having made myself master of the blood by means of a tourniquet, I made an incision in the integuments without touching, in any way, the aneurismal sac; I then sought for the artery, which I found situated below the nerve, which is not common. I took every precaution in separating it from this, and having lifted it upon a hook, I ligatured it as near to the tumour as possible. The artery having been tied, I loosened the tourniquet; when a small muscular branch, which I had divided in dissecting the vessel bled, and compelled me immediately to tighten the tourniquet and to tie the artery again a little higher up. The tourniquet being loosened I saw no more bleeding, nor any pulsation in the tumour. I then applied the proper dressings and a bandage." 220.

The patient was bled from the other arm on the day of the operation, and the venæsection was repeated three times during the after-treatment. On the following morning, a distinct pulse was felt in the wrist of the aneurismatic arm—a circumstance, says M. Anel, "which gave me ground to hope, seeing myself so well seconded by nature, which in a single night had made a new channel by which the blood was conveyed from the arm to the fore-arm, and even to the extremity of the hand.

"Having then no fear of mortification, I devoted the whole of my attention to prevent fever and hemorrhage. I left the first dressing seventy-two hours without touching it. The third day I dressed the wound with a compound digestive. I also fomented the arm with red wine, to which I added a quarter of camphorated spirits; and only dressed the patient once in the twenty-four hours. By continuing this plan the cure was happily accomplished without any accident. The first ligature separated on the 17th day of February, 1710; and the second, on the 27th of the same month, without the supervention of the least hemorrhage. On the 1st of March, in the same year, this friar not only left his room, but went even to the church of Saint Laurent, in Damasco, which was at the distance of an Italian mile from his convent. On the 5th of March, the wound was perfectly cicatrized. A month after the operation had been performed, the patient used his arm just as before the accident, without the least weakness or pain. The pulsation of the aneurismal tumour disappeared as soon as the artery was tied above it, which ought to happen in accordance with the laws of the circulation of the blood. However, in accordance with the general opinion, this pulsation should have continued; because it is said, that when the operation is performed, the artery should be tied as well below as above, on account of the anastomoses of the branches of the arteries; by which the blood passes into that portion of the trunk that has given way; and that the blood having entered the trunk, refills the tumour, and causes bleeding by returning from below. If all this were true, the pulsation should have continued, and this tumour would not have disappeared unless the ligature had been applied below it; nevertheless, without having done so, the tumour collapsed in such a way that it would have been impossible to have ascertained the spot where the aneurism had existed."

His remarks on the operation are well worthy of notice; they shew that he was far a-head, not only of his contemporaries, but of the next generation also, in the knowledge of the true principles that should guide the surgeon in the treatment of Aneurism.

"With regard to the mode of doing the operation, I performed it in a different way to what authors describe, which I have seen good surgeons adopt, and which I have myself had recourse to several times; for instead, as is customary, of applying the ligature above and below the aneurism, I only practised it above. Besides, the aneurismal sac is usually opened, but I did not touch it at all: not doubting but that the blood contained in it would be dissipated, being at liberty to pass on towards the extremity; that the sac, being once empty, would not fill again; that the layers of membrane that formed it would not fail to collapse; and that thus the tumour would disappear: all which happened as I had thought.

"In this way the operation was less tedious, and much less painful; besides, my incision was not half the usual length, hence there was a smaller cicatrix. If I had opened the aneurismal sac, and tied it below, the cicatrix could not have been exactly at the bend of the arm, and might have prevented extension being perfectly performed; which I have seen happen to several patients, who have been maimed by this operation, in consequence of the situation and extent of the cicatrix." 223.

Is it not surprising that the judicious practice of Anel was not imitated by others, after they heard of the success that had attended it? But such is the force of prejudice and long-established usage, that few men have the gift of seizing upon an improvement, when it is first proposed. We find indeed that Heister (in 1744) suggests the application of Anel's operation to cases of popliteal aneurism; for we find him saying:—

"Nevertheless, as this operation can be performed on the arm, as the President and many other modern surgeons are well aware of, I do not think it is impracticable in this situation, if it be done in the same way as in the arm. The tourniquet having been adjusted and tightened, and the patient laid prone on the belly, the popliteal artery should be sought for, the parts covering it having been divided, and a thread being passed round the vessel, above the rupture, should be tied so tight, that, on the tourniquet being slacked, no blood may escape from the seat of disease; the wound must afterwards be dressed and healed, in the same way as in an aneurism of the arm." 233.

The idea, however, seems never to have been carried into effect, until the time of Dessault; who, in a case of popliteal aneurism, tied the artery immediately above the tumor, and effected a cure; although the tumor subsequently gave way, and the patient died several months afterwards, of the consequences of his disease, complicated with caries of the tibia. This was in 1785.

Such little progress had the surgery of the arteries made in the half-century after Anel's operation, that we find Bromfield—who was surgeon to Her Majesty and to St. George's Hospital—uttering the following opinion*:—

"The injecting the parts of dead bodies having discovered that, in particular subjects, the branches sent off from the principal trunks of arteries have, now and then, formed anastomoses with other branches that have gone off lower down; and from observing, likewise, that after the operation for the aneurism has been performed, where the artery had been unfortunately wounded in blood-letting, that the lateral vessels have become dilated, in time, sufficiently to carry on the circulation in that limb. From these observations the most extravagant propositions have been by some suggested, viz., that the tying of the principal trunks of the arteries of any of the extremities, when wounded, may be done with a fair prospect of preserving the limb. I once saw an attempt of this kind in a true aneurism, situated in the ham, on which I shall make no further remark than that the patient died; and I dare believe that the embarrassments which occurred, as well as accidents in the operation, will deter the operator from making a second attempt, should a similar case offer for his assistance." 348.

Six years later, the celebrated Pott, in his Remarks on the necessity and propriety of Amputation in certain cases, expressly tells us that the operation then employed for the treatment of popliteal Aneurism was almost always unsuccessful, and uses these remarkable words: "Nor have I ever seen any other operation than that of amputation which has preserved the life of the patient."

In the same year, Wilmer writes thus:†

"With regard to the case of which we are now speaking, (the aneurism of the popliteal artery,) there is not, that I know, a single case upon record, where the operation for the aneurism hath succeeded. It hath been done several times within these few years, in our public hospitals, but I have not heard of any one case where it answered the intended purpose." 358.

Murray‡ is the first that we find alluding to any experiments on animals, with the view of ascertaining the effects of tying the femoral artery.

"The experiments of George Martin, of Gooch, and of Revans, on a dog, whose femoral artery was tied in the middle of its course, together with the vein and nerve, the animal being perfectly cured by the twelfth day, without the least injury to the limb, confirm me in my opinion that it is so decreed throughout the whole of the animal economy, by the admirable goodness and wisdom of the allwise Providence, that the trunks of those arteries that are, on account of their

---

* Surgical Observations and Cases. London, 1773, p. 303.

† Cases and Remarks in Surgery, &c. London, 1779.

‡ In aneurysmata femoris Observationes, &c. &c. Upsaliæ, 1781.

superficial situation, exposed to external violence, have, as it were, a vicarious action with others that are more deeply seated, and which are most closely connected with them. Numerous experiments that have been performed, both in the upper and lower limbs, favour this opinion. Is it then inconsistent to attribute to the thigh that degree of perfection which it certainly requires on account of its great bulk?" 361.

It was in December, 1785, that John Hunter performed the first operation of tying the femoral artery, about the middle of its course on the inner part of the thigh, for the cure of a popliteal aneurism. He had himself experienced the danger of tying the vessel near the seat of the disease, and justly referred the unsuccess of this operation to the morbid state of the arterial parietes. It was no random guess, but the philosophical deduction from practical observation, that led him to propose the change. How admirably he anticipates the result of the operation is apparent from the following passage in the report published by Sir Everard Home at the time, in the London Medical Journal:—" Mr. Hunter being too much engaged to permit his taking that task upon himself."

" The force of the circulation being thus taken off from the aneurismal sac, the cause of the disease would, in Mr. Hunter's opinion, be removed; and he thought it highly probable that, if the parts were left to themselves, the sac, with the coagulated blood contained in it, might be absorbed, and the whole of the tumor removed by the actions of the animal economy, which would consequently render any opening into the sac unnecessary." 374.

As everything connected with this operation—the dawn of a better state of things, and the parent-cause of all the brilliant achievements of modern surgery in the treatment of Aneurisms—cannot fail to be interesting, we shall notice a few particulars. No fewer than four ligatures were employed.

" Having disengaged the artery from its lateral connexions by the knife, and from the parts behind it by means of the end of a thin spatula, a double ligature was passed behind it by means of an eyed probe, and the artery tied by both portions of the ligature, but so slightly as only to compress its sides together; a similar application of ligature was made a little lower; and the reason for passing four ligatures was to compress such a length of artery as might make up for the want of tightness, as he chose to avoid great pressure on the vessel at any one part. The ends of the ligatures were carried directly out at the wound, the sides of which were now brought together and supported by sticking plaster and a linen roller, that they might unite by the first intention." 375.

Everything went on favourably until the ninth day, when there was a considerable discharge of blood from the part where the ligatures passed out—the rest of the wound having healed. A tourniquet was applied; the bleeding stopped, and did not return when the compression was removed a few hours afterwards. The head of a roller was now placed upon the wound in the direction of the artery, and over that the tourniquet, which was not tightened more than was thought sufficient to take off the impetus of the blood in that portion of the artery. On the 15th day, some of the ligatures came away. Six weeks after the operation, the patient left the hospital; the tumor in the ham being considerably reduced, and firmer to the touch. On March 8th, the wound, which had cicatrised, broke out again, and the patient returned into the hospital. A month afterwards, some remaining threads came away, and an inflammation appeared on the upper part of the thigh. In May, a small abscess broke at some distance from the old cicatrix. Several small threads were, at different times, discharged at the old sore. In the beginning of July, a piece of ligature, about an inch in length, came away. A week afterwards the patient left the hospital, in every respect well: no tumor remained in the ham. He died in the following year from remittent fever, and an opportunity was had of examining the state of the arteries in the leg that had been operated upon: we must afford room for the report of the examination.

No tumor was perceptible by the eye in the ham of the affected limb; but to the touch there was a solid lump or swelling, filling up the hollow between the two condyles of the thighbone. The arteries and veins having been carefully injected, the following appearances were discovered on dissection.

"The femoral artery was impervious from the giving off the profunda, as low as the part which was included in the ligature, and at this part there was an ossification, for about an inch and a half along the course of the artery, of an oval form, the run of which was solid, becoming thinner towards the centre, and not bony but only ligamentous; below this part the femoral artery was pervious down to the aneurismal sac, and contained blood, but did not communicate with the sac itself, having become impervious just at the entrance.

"What remained of the aneurismal sac was somewhat larger than a hen's egg, but more oblong, and a little flattened, extending along the side of the artery below for some way; the blood pressing with greater force in that direction, and distending that part, so as in some measure to give the appearance of a separate bag. The sac was perfectly circumscribed, not having the smallest remains of the lower orifice from the popliteal artery." 382.

A solid coagulum of blood, composed of concentric lamellæ, was found adherent to the inner surface of the aneurismal sac.

Mr. Home, after remarking how correctly Mr. Hunter had predicted, prior to the operation, the effects which tying the vessel above the seat of the disease would induce, proceeds to observe that the important conclusion to be drawn from the history of this case is, "that the simply taking off the force of the circulation from the aneurismal artery is sufficient to effect a cure of the disease, or at least to put a stop to its progress, and leave the parts in a state from which the actions of the animal economy are capable of restoring them to a natural one." He adduces, in support of this view, a most interesting case, (that occurred in the practice of Mr. Ford of Golden Square), where an aneurismal swelling in the upper part of the thigh was seized with inflammation and threatening mortification.

"While in this state, the pulsation, before very evident in every part of the tumor, was no longer to be felt, nor even in the artery immediately above it, so that the steps preceding mortification had certainly taken place, the blood in the artery above having coagulated. And this circumstance was sufficient to prevent the absolute mortification coming on; for the artery above becoming impervious, put a stop to the dilatation of the sac and all its consequences.

"From the time the pulsation stopped, the swelling and inflammation subsided, although exceedingly slowly, and the tumor diminished, becoming more firm and solid, and at the time of writing this paper is very much reduced in size, and to the feel resembles that found in the ham of the patient who is the subject of this paper." 384.

The first surgeon, who seems to have adopted the Hunterian operation, was Mr. Birch of St. Thomas's Hospital. The case was an aneurism of the *femoral* artery, extending within two inches of Poupart's ligament, and occupying two-thirds of the thigh. The artery was exposed at about two inches or so below the groin, and two ligatures were passed round it; the lower one only being tied. On the eleventh day after the operation, the aneurismal tumor burst, and discharged serum and grumous blood; the patient died in the course of the evening. On *dissection*, the artery was found to be ulcerated at the seat of the ligature; this had been applied around the vessel at rather less than half an inch below the giving off of the *profunda* branch, and nearly two inches above the upper end of the aneurismal swelling. The integuments over the middle of the swelling were mortified, and the blood within it was fluid and putrescent.

The reader will observe that this was a most unfavourable case to test the value of the new operation: the ligature was applied not only too near the seat of the disease, but also immediately below the giving off of a large branch.

So little did some of the leading surgeons of that day appreciate the importance of Hunter's method, that we find Pott performing the following operation upwards of a twelvemonth after its introduction.

"The aneurism was in the ham, and the operation was performed by Mr. Pott in the following manner: An incision was made above the tumor, through the integuments, in the direction of the thigh between the two hamstrings, for about five inches in length; he then dissected down to the vessels at the upper end of the incision, which being there deep-seated, it proved both tedious and difficult. Having come down to the vessels, a double ligature was passed, and the two portions tied separately at nearly half an inch distance. The depth of the incision made it difficult for any but the operator, and those immediately assisting him, to see what was included in the ligature, and no doubt was made at the time of its being anything but the artery. The wound was dressed up in the common way.

"The second day after the operation a pulsation was felt in the tumor, which afterwards enlarged so much that Mr. Pott amputated the limb.

"On dissection, the aneurism did not appear to be in the artery which was included in the ligature, but was supposed to be in an anastomosing branch." 384.

A case operated upon by Mr. Lynn of the Westminster Hospital, very nearly after the plan adopted by Hunter, proved perfectly successful. Not so one at St. Thomas's Hospital, under the care of Mr. Cline, where the following clumsy mode of tying an artery was practised.

"Mr. Cline made a longitudinal incision on the anterior part of the thigh, and laid bare the artery, passed, by means of a tin instrument, a double tape, about one inch broad, behind the artery, the two pieces of tape lying one over the other; the piece of tin which conducted the tape was cut off, and a cork, nearly an inch long, was laid upon the artery, and confined to its situation by means of the upper tape, producing in this way a sufficient pressure upon the vessel included between the ligature and cork to stop the circulation, and consequently the pulsation in the tumor in the ham: the other portion of tape was left loose. The intention of securing the artery in this way was to compress the sides of the vessel together, and produce an union without ulceration.

"The patient went on very well, and the ninth day the tapes were removed, and everything seemed to be going on very favorably, when the patient was attacked by a fever (which was supposed to be caught from another patient in the same ward), of which he died. Upon examining the state of the limb after death, it was found that ulceration had taken place through the whole extent of the artery included in the tape, and sinuses were formed both upwards and downwards, in the course of the thigh, to some distance." 399.

In Mr. Hunter's second operation, the femoral artery and vein "were included in *one* strong ligature, sufficiently tight to prevent the pulsation in the one, without injuring the coats of the vessels."

The ligature came away on the fourteenth day. In consequence of hæmorrhage from the wound a few days afterwards, the artery was laid bare and tied a little higher up. But the hæmorrhage returned, and the patient died on the sixth day after the second operation, and the twenty-sixth after the first.

In the *third* case, also, only one ligature was employed: it came away on the fourteenth day. The patient recovered perfectly.

In the *fourth* operation, "the vein was not included in the ligature, but in other respects it was similar to the former." The man quite recovered.

The *fifth* case, also, was successful, the artery alone having been included in a single strong ligature.

M. Chopart was the first who adopted the Hunterian method in France (March 1792). The case proved unsuccessful, the foot and leg becoming gangrenous.

A few days afterwards, M. Deschamps performed the same operation. He made use of two large ligatures, each consisting of a skein of thread. There seems to have been a great deal of bungling on the part of the surgeon in tying the artery. In spite of some mishaps, however, the case ultimately did well, and the patient was perfectly cured.

After relating several other cases, M. Deschamps enters upon a dissertation with regard to the relative merits of the Hunterian operation, and the old one by incision of the sac. The conclusion, to which he arrives, is given in these words:

"It follows, from what we have said, that if the facts, especially those that have occurred under our own observation, are not as yet sufficiently numerous to justify us in giving a decided preference in favour of Hunter's operation over that by incision of the sac, yet they are sufficient to authorize the surgeon to have recourse to Hunter's plan, when the circumstances of the case are favorable to success." 467.

We need not say what has been the result of surgical experience during the last half-century. If our great countryman had done nothing else for science, his operation for aneurism alone would have secured him immortality. Many of the modern French writers have enviously claimed for Anel the praise of the operation, and indulge their national pride by calling it Anel's method. Let us hear what their own countryman Deschamps, in 1797, has written on the subject.

"It may be seen, by what I have just stated concerning these different modes of operating for aneurism, that Anel, as well as Guillemeau, employed the ligature above the tumor, but that their operations differed, inasmuch as Anel did not meddle with the tumor itself; and that Hunter's plan differs from Anel's, inasmuch that the latter, as well as Guillemeau, ligatured the vessel *immediately* above the aneurism, whilst Hunter tied it at a *considerable distance above this.* Hunter has employed this plan since 1786, and it appears to be pretty successful in his hands." 418.

